# Characterization of large genomic deletions in the *FBN1 *gene using multiplex ligation-dependent probe amplification

**DOI:** 10.1186/1471-2350-12-119

**Published:** 2011-09-21

**Authors:** Larissa V Furtado, Whitney Wooderchak-Donahue, Alan F Rope, Angela T Yetman, Tracey Lewis, Parker Plant, Pinar Bayrak-Toydemir

**Affiliations:** 1 Department of Pathology, University of Utah Health Science Center, Salt Lake City, UT, 84108, USA; 2 ARUP Institute for Clinical and Experimental Pathology, Salt Lake City, UT, 84108, USA; 3 Department of Pediatrics, Division of Medical Genetics, University of Utah Health Science Center, Salt Lake City, UT, 84108, USA; 4 Department of Pediatrics, Division of Cardiology, Primary Children's Medical Center, Salt Lake City, UT, 84112, USA

## Abstract

**Background:**

Connective tissue diseases characterized by aortic aneurysm, such as Marfan syndrome, Loeys-Dietz syndrome and Ehlers Danlos syndrome type IV are heterogeneous and despite overlapping phenotypes, the natural history, clinical manifestations and interventional course for each diagnosis can be quite unique. The majority of mutations involved in the etiology of these disorders are missense and nonsense mutations. However, large deletions and duplications undetected by sequencing may be implicated in their pathogenesis, and may explain the apparent lack of genotype-phenotype correlation in a subset of patients. The objective of this study was to search for large pathogenic deletions and/or duplications in the *FBN1, TGFβR1*, and *TGFβR2 *genes using multiplex-ligation dependent probe amplification (MLPA) in patients with aortopathy, in whom no mutations in the *FBN1, TGFβR1*, and *TGFβR2 *genes were identified by sequencing.

**Methods:**

The study included 14 patients from 11 unrelated families with aortic aneurysm. Of those, six patients (including 3 first-degree relatives), fulfilled the revised Ghent criteria for Marfan syndrome, and eight had predominantly aortic aneurysm/dilatation with variable skeletal and craniofacial involvement. MLPA for *FBN1, TGFβR1*, and *TGFβR2 *was carried out in all patients. A 385 K chromosome 15 specific array was used in two patients with a deletion of the entire *FBN1 *in order to define its size and boundaries.

**Results:**

We identified two novel large deletions in the *FBN1 *gene in four patients of two unrelated families who met clinical diagnostic criteria for Marfan syndrome. One patient was found to have a *FBN1 *deletion encompassing exons 1-5. The other three patients had a 542 Kb deletion spanning the whole *FBN1 *gene and five additional genes (*SLC24A5, MYEF2, CTXN2, SLC12A1, DUT*) in the chromosome 15.

**Conclusions:**

Our findings expand the number of large *FBN1 *deletions, and emphasize the importance of screening for large genomic deletions in connective tissue disorders featuring aortopathies, especially for those with classic Marfan phenotype.

## Background

Thoracic aortic aneurysm with dissection is the most common fatal condition involving the aorta [1], and can be syndromic, familial nonsyndromic or sporadic. Mutations in genes related to the structure and function of the aortic wall, such as *MYH11 *on chromosome 16p12.2-13.3 [2,3], *ACTA2 *on chromosome 10q23-24 [4], *SLC2A10 *on chromosome 20q13.1 [5], *NOTCH1 *on chromosome 9q34-35 [6], *TGFβR1 *on chromosome 9q33-34 [7], and *TGFβR2 *genes on chromosome 3p24-25 [8] have been linked to non-syndromic familial forms of thoracic aortic aneurysm [reviewed in 9]. Syndromic connective tissue diseases featuring aortic aneurysm and dissection comprise a heterogeneous group of genetic diseases, including Marfan syndrome (MFS; OMIM # 154700), Loeys-Dietz syndrome (LDS; OMIM # 609192), Ehlers Danlos syndrome type IV (EDS IV; OMIM # 130050), and familial thoracic aneurysm syndrome (TAAD; OMIM# 132900). Recently, a new syndrome presenting with aneurysms, dissections and tortuosity throughout the arterial tree and mild craniofacial features, early-onset osteoarthritis and skeletal and cutaneous anomalies was described to be associated with mutations in the *SMAD3 *[10]. These conditions have broad spectrum of phenotypic expression and overlapping phenotype, although their natural history and interventional course can be quite unique. Making the timely and correct diagnosis in individuals with connective tissue disorders with aortopathy is crucial to obtaining appropriate surveillance and interventions aimed at preventing the significant morbidity and mortality associated with these diseases. In this setting, molecular testing is a valuable adjunct to clinical assessment, especially in cases with non-classical phenotypes. The majority of syndromic connective tissue featuring aortopathy are caused by missense, nonsense or splice site mutations [11]. A few reports of large genomic deletions involving single and multiple exons of the *FBN1 *gene [12-17] as well as whole-*FBN1 *deletions [18-22] are available, although no large *FBN1 *duplications have been reported to date. Because of that, full gene sequencing is commonly the initial approach for the diagnosis of these patients. However, sequencing does not detect large exonic deletions or duplications unless quantitative methods are applied, which allegedly could explain the lack of genotype-phenotype correlation in some patients.

This study was undertaken to search for large pathogenic deletions and/or duplications in the *FBN1, TGFβR1*, and *TGFβR2 *genes using multiplex-ligation probe amplification (MLPA) in 14 patients with clinical diagnosis of aortopathy in whom no mutations in the *FBN1, TGFβR1*, and *TGFβR2 *genes were identified by sequencing.

## Methods

### Subjects

Informed consent was obtained from patients and relatives using University of Utah and Primary Children's Medical Center Institutional Review Board-approved protocols. The study included 14 patients [10 males, 4 females; mean age: 21 years (age range: 2- 43 years)] from 11 unrelated families, who had clinical diagnosis of aortic dilatation, aneurysm or aortic dissection and negative molecular analysis of the *FBN1, TGFβR1*, and *TGFβR2 *genes by sequencing. In families with more than one affected individual, the result from mutational analysis was extrapolated to untested relatives. Of those, 6 patients (including 3 first-degree relatives), fulfilled the revised Ghent criteria for diagnosis of Marfan syndrome [23], and 8 had predominantly aortic aneurysm or dilatation with variable skeletal and craniofacial involvement (Table 1). Data on the clinical phenotypes of patients were collected from medical records and during physical examinations during their visit to the Medical Genetics and Cardiology clinics at Primary Children's Medical Center.

**Table 1:** Summary of clinical profile of patients

	1	2*	3*	4*	5	6	7	8§	9§	10	11	12	13	14
**Gender**	**M**	**F**	**M**	**M**	**M**	**F**	**M**	**M**	**F**	**M**	**F**	**M**	**M**	**M**

**Age (years)**	27	42	15	12	42	19	12	30	2	15	25	18	14	27

**Aortic**														
Root dilatation (Z ≥ 2)	+	+	+	+	+	+	+	+	+/-	+	-	+	+	+
Ao root surgery	-	+	-	-	+	-	-	-	-	-	-	-	-	-
Aneurysm	-	+	-	-	-	-	-	-	-	+	+	-	-	-
Dissection					-	-	-			-	+	-	-	-
Tortuosity					+	-	-			-	-	-	-	-

**Cardiac**														
Mitral valve prolapse	+	-	-	-	-	+	-	-	-	-	-	+	-	+
Valve (other)	+	+	+	+	-	+	-	+	-	-	-	+	-	-
Cardiac (other)	-	-	-	-	-	-	-	-	+	-	+	-	-	-

**Ophthalmologic**														
Ectopia lentis	-	-	+	-	-	-	-	-	-	-	-	-	-	-

**Skeletal**														
Pectus carinatum	+	+	-	-	+	+	-	-	-	+	-	-	-	-
Pectus excavatum	+	+	+	+	-	-	+	-	-	-	-	-	-	+
Reduced US/LS	+	+	+	+	+	+	-	-	-	-	-	-	-	+
Increased arm/height	-	-	-	-	+	+	+	-	-	-	-	-	-	-
Scoliosis	-	-	-	-	+	-	-	-	-	+	-	-	-	-
Thoracolumbar kyphosis	-	-	-	+	-	-	-	-	-	-	-	-	+	+
Protrusio acetabuli	-	-	-	-	-	-	+	-	-	+	-	-	-	-
Pes planus	-	-	-	-	-	-	-	-	-	-	-	-	-	-
Hindfoot deformity	+	-	+	+	-	+	-	-	-	-	-	-	+	+
↓ elbow extension					+		-	-		-	-	-		
Wrist/thumb signs														
Dural ectasia														

**Craniofacial**														
Hypertelorism	-	-	-	+	-	-	-	+	-	-	-	-	-	-
Bifid/broad uvula	-	-	-	-	-	-	-	+	-	-	-	-	-	-
Palate anomaly	-	+	-	+	-	-	-	+	-	-	-	-	-	-
Microretrognathia	+	-	-	-	-	-	-	+	-	-	-	+	-	-
Dolicocephalia	-	-	-	-	-	-	-	-	-	-	-	-	-	-
Craniosynostosis	-	-	-	-	-	-	-	-	-	-	-	-	-	-
Other	-	-	-	-	-	-	-	-	-	-	-	-		

**Skin**														
Striae	+	-	+	-	+	+	-	-	-	-	-	-	-	+
Thin and velvety skin	-	-	-	-	-	-	-	-		-	-	-	-	-
Easy bruising	-	-	-	-	-	-		-			-	-	+	-

**Family History**	+	+	+	+	+	-	+	+	+	+	+	+	-	-

**Ghent**	+	+	+	+	+	-	-	-	-	-	-	-	-	+

***FBN1 *sequencing**	Neg	Neg	Neg	Neg	Neg	Neg	Neg	Neg	Neg	Neg	Neg	Neg	Neg	Neg

***TGFβR1/2 *sequencing**	Neg	Neg	Neg	Neg	Neg	Neg	Neg	Neg	Neg	Neg	Neg	Neg	Neg	Neg

Abbreviations:

* Related patients: patient 2 = mother; patients 3 and 4 = sons.

§ Related patients: patient 8 = father; patient 9 = daughter.

+: present; -: absent; blank spaces: information not available

Neg: Negative

### Multiplex Ligation-dependent Probe Amplification (MLPA)

Genomic DNA was extracted from peripheral blood using standard procedures (Gentra Puregene Blood Kit, Qiagen, Valencia, CA). MLPA assays were performed in duplicate according to the manufacturer's instructions [24]. The P065 and P066 Marfan Syndrome MLPA kits (MRC-Holland, Amsterdam, The Netherlands) [25,26] that contain probes for 54 of the 66 *FBN1 *exons and 7 *TGFβR2 *exons, were used to detect deletions or duplications in *FBN1*. The P065 and P066 probemixes do not contain probes for *FBN1 *exons: 1, 11, 12, 21, 23, 28, 33, 38, 40, 49, 52, 60, and for *TGFβR2 *exon 2. P148 Loeys-Dietz MLPA kit (MRC-Holland, Amsterdam, The Netherlands) [27] was used to detect deletions or duplications in *TGFβR1 *and *TGFβR2 *genes. The P148 probemix contains probes for all exons of the *TGFβR1*and *TGFβR2 *genes with the exception of *TGFβR2 *exon 2. Amplification products from each MLPA assay were separated by capillary electrophoresis on an ABI 3100 Genetic Analyzer (Life Technologies, Carlsbad, CA, USA) and results were analyzed using GeneMarker^® ^software version 1.6 (SoftGenetics, State College, PA, USA). Deletions and duplications of the targeted regions were detected when the height ratios of the fluorescent peaks were lower or higher than the normal height ratio range of 0.7-1.4, respectively.

### Sequencing

The coding exons and flanking intronic regions for *TGFβR1, TGFβR2 *and *FBN1 *were PCR-amplified for all patients. Primers sequences are available upon request. Amplicon fragments were bi-directly sequenced with universal M13 primers using the Big Dye^® ^Terminator v3.1 cycle sequencing kit and an ABI 3730 DNA Analyzer (Life Technologies, Carlsbad, CA, USA). Sequences were compared to the *TGFβR1 *and *TGFβR2 *reference sequences (NM_004612.2 and NM_003242.5, respectively) and to the *FBN1 *reference sequence (NM_000138.4) using Mutation Surveyor software version 3.01 (SoftGenetics, State College, PA, USA).

In our Institution, we sequenced the *TGFβR1 *and *TGFβR2 *from seven patients, and the *FBN1 *gene from two patients. The remaining cases were sequenced at the Connective Tissue Gene Test (CTGT) Laboratory.

### Array-CGH 385 K Chromosome 15 Specific Array

DNA from peripheral blood leukocytes from patients 2 and 3 was extracted using standard techniques (Gentra Puregene Blood Kit, Qiagen, Valencia, CA). One μg of patients' DNA was labeled with 5'- Cy3 tagged nanomers while the female control was labeled with Cy5 nonamers (Roche NimbleGen, Madison, WI). After purification by isopropanol precipitation, 6 μg each of labeled patients and reference DNA were combined. The mixture was hybridized to a NimbleGen 385 K Chromosome 15 Specific Array for 16 hours at 42 degrees in a MAUI Hybridization System (BioMicro Systems). The array was then washed according to the manufacturer's recommendation (Roche NimbleGen, Madison, WI) and immediately scanned at 5 micron resolution. After scanning, fluorescence intensity raw data was extracted from the scanned images of the array using NimbleScan v2.5 software. For each of the spots on the array, normalized log2 ratios of the Cy3-labeled patient sample vs the Cy5-reference sample were generated using the SegMNT program. The data was visualized with Nexus v5.1 software (Biodiscovery, El Segundo, CA).

## Results

**Figure 1:**
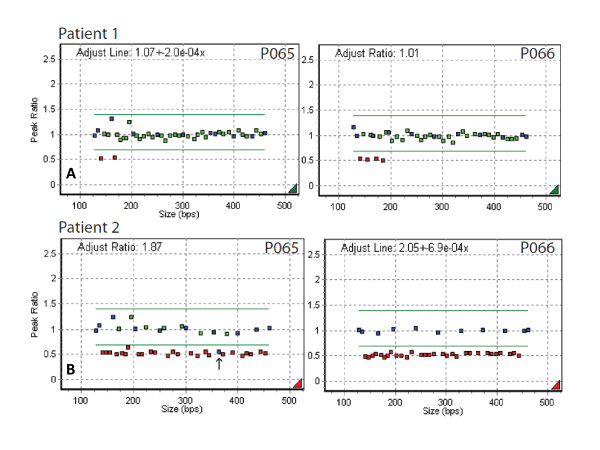
***FBN1 *deletions observed in cohort**. In panel A, MLPA results for patient 1 show the deletion of exons 1-5 of the *FBN1 *gene. In panel B, MLPA results for patient 2 with the deletion encompassing the whole *FBN1 *gene and an additional control probe (black arrow), located on the *DUT *(*deoxyuridine triphosphatase*) gene, 301 Kb upstream from *FBN1 *exon 1, on chromosome 15q15-q21.1. The same deletion was also found in patients 3 and 4 (data not shown).

In our study, we identified two novel large deletions in the *FBN1 *gene in four patients of two unrelated families who met clinical diagnostic criteria for Marfan syndrome. Of those, a novel *FBN1 *deletion encompassing exons 1-5 (Figure 1, Panel A) was identified in a 27-year-old male (patient 1; Tables 1 and 2) with dilated aortic root (3.99 cm, z-score of 3.76), mild tricuspid and mitral valve prolapse, tricuspid valve insufficiency, height 201 cm (6' 8") arm span 205.5 cm (6'10"), arachnodactyly, joint hypermobility, positive thumb and wrist signs, combined pectus carinatum/excavatum deformity of the anterior chest wall, and marked diffuse striae over the lower back and hips. Family history was remarkable for a diagnosis of Marfan syndrome in the mother, who died secondary to complications from surgery for aortic root dissection years prior, and one 30-year-old affected brother, who was unavailable for molecular testing.

**Table 2:** MLPA Assay Results for *FBN1, TGFβR1, TGFβR2 *and *COL3A1*

Subjects	Deletion/Duplication Results
Patient 1	***FBN1 *exons 1-5 deletion**
Patient 2	***FBN1 *full gene deletion**
Patient 3	***FBN1 *full gene deletion**
Patient 4	***FBN1 *full gene deletion**
Patient 5	Normal
Patient 6	Normal
Patient 7	Normal
Patient 8	Normal
Patient 9	Normal
Patient 10	Normal
Patient 11	Normal
Patient 12	Normal
Patient 13	Normal
Patient 14	Normal

**Figure 2:**
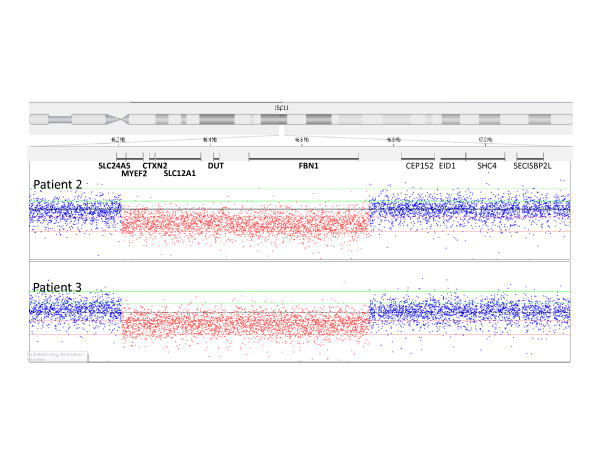
**NimbleGen 385 K Chromosome 15 Specific Array showing a 542 kb region of chromosome 15 loss involving 6 genes (SLC24A5, MYEF2, CTXN2, SLC12A1, DUT, and FBN1), which was identical in patients 2 and 3**.

The other large deletion was found in a family of a mother and two sons (patients 2, 3, and 4, respectively; Tables 1 and 2), who harboured a deletion of the entire *FBN1 *gene. In those cases, all *FBN1 *probes and one control probe, located on the *DUT *(deoxyuridine triphosphatase) gene which is 301 Kb upstream from *FBN1 *exon 1 on chromosome 15q15-q21.1, were deleted (Figure 1, Panel B). A NimbleGen 385 K chromosome 15 specific array performed on patient 2 and 3 to refine the deletion size and boundaries revealed a 542 Kb deletion spanning bases chr15:46,208,030-46,750,218/Hg 18 Genome Build. This deleted region included 5 additional genes: *SLC24A5, MYEF2, CTXN2, SLC12A1*, and *DUT *(Figure 2). Clinically, patient 2 (mother), had a history of aortic valve replacement, and was status post Bentall procedure with a porcine valve in the setting of aortic dissection in 2005. She also had an abdominal aortic aneurysm. Skeletal involvement was characterized by a height of 190 cm (6'4"), arm span 197 cm (6'6"), pectus carinatum, arachnodactyly, and joint hypermobility. Patient 3 had aortic root (3.72 cm, z-score 3.22) as well as ascending aortic (2.69 cm) dilation, height 193.5 cm (6'5"), arm span 198.2 cm (6'7"), pectus excavatum deformity, positive thumb sign, positive wrist sign, joint hypermobility, and striae over both legs. Ocular examination showed bilateral ocular lens subluxation. His younger brother (patient 4) had a dilated aortic root (3.7 cm of diameter, z-score of 5.7), bicuspid aortic valve, pectus excavatum deformity, arachnodactyly, significant joint laxity with positive thumb sign and positive wrist sign, marked pes planus with downward deviation of the medial malleolus, height greater than the 99th percentile for age, an arm span at 105% of height, hypertelorism, and high narrow palate.

In all other patients, including a 27-year-old male with classical Marfan syndrome phenotype (patient 14), no large deletions or duplications of the *FBN1, TGFβR1*, and *TGFβR2 *genes were identified.

## Discussion

Only a few large *FBN1 *deletions involving single and multiple exons have been reported and have related to the most severe MFS phenotypes. Among those, deletions of exons 13-49 in mosaic [12]; deletion of whole exon 33 [12]; inframe deletions of exon 2 [17], exons 42-43 [13], exon 52 [17] and exons 60-62 [16]; deletions spanning exon 1 [14], in-frame deletion of exons 44-46 [13] and an out-of-frame deletion of exons 58-63 [15] were previously described. Increased phenotypic severity does not particularly apply to patients with complete *FBN1 *deletions [18-22]. Recently, Hilhorst-Hofstee et al. (2010) [18] published a series of 10 patients with whole-*FBN1 *gene deletion presenting with phenotypes ranging from mild features of MFS to the classical MFS phenotype. In five of their patients, deletions extended beyond the *FBN1 *gene, spanning 1-9.4 Mb, including 9-46 genes. All of those deletions encompassed the same genes located upstream to the *FBN1 *as the ones involved in patient 2 and 3 of our cohort, although the breakpoints, size and position of the deletions differed between the two studies. Despite the additional deletion of five contiguous genes (*SLC24A5, MYEF2, CTXN2, SLC12A1, DUT*), our patients: 2, 3 and 4 had no other features than those that can be attributed to the deletion of *FBN1*. The same was observed in two patients from Hilhorst-Hofstee et al. [18] found to have deletion of 9 genes including *FBN1*. These findings not only support the role of haploinsufficiency of *FBN1 *in the pathogenesis of MFS, but also suggest that the function of *SLC24A5, MYEF2, CTXN2, SLC12A1, DUT *may not be impaired by complete loss of an entire allele.

Three additional patients with 15 q deletions that included and extended beyond *FBN1 *were previously reported [19,21,22]. Their phenotype was characterized by marfanoid features predominantly affecting the skeletal system, with absent or mild aortic involvement. Psychomotor retardation and microcephaly was also described in two of those patients for whom the sizes and breakpoints of their 15 q deletions were unavailable [21,22]. The 16-year-old female described by Faivre et al. [19] harboured a 15q21.1q21.2 micro deletion of 2.97 Mb that encompassed the entire *FBN1 *and 12 additional genes, including the same genes as the ones involved in patient 2 and 3 of our cohort, although the breakpoints, size and position of the deletions differed between the two studies. As for our patients: 2, 3 and 4, no other features than those that can be attributed to the deletion of *FBN1 *were observed in their patient.

An important consideration is that all patients in our study, who harboured a *FBN1 *deletion, fulfilled the Ghent criteria for MFS with major manifestations in the skeletal and cardiovascular systems. This is especially remarkable given the relatively young ages of patient 3 and 4, and suggests that the increased severity of MFS due to large *FBN1 *deletions may overcome the incomplete expression of MFS phenotype known to occur in children. In addition, the degree of severity and the distribution of the major manifestations differed among affected individuals, in keeping with the clinical variability seen in MFS, which in our patients, could be due to variable *FBN1 *expression from the normal allele.

In our cohort, cases with non-classical phenotypes showed no large deletions and duplications in the genes targeted by MLPA. Technical limitations of available testing techniques, genetic heterogeneity and varied pathogenetic mechanisms involved in each of the connective tissue diseases with aortopathy may account for the lack of genotype-phenotype correlation in our subset of patients. For instance, commercially available kits for analysis of the *FBN1 *gene (P065 and P066) contain probes for 54 of the 66 *FBN1 *exons; and the probemix for *TGFβR2 *(P148) does not contain probes for exon 2. Therefore, a deletion or duplication of non-tested exons cannot be excluded leading to probable underreported deletions or duplications in this gene. Mosaicism and copy-number neutral rearrangements may also not be detected by MLPA. In addition, current molecular genetic testing of *FBN1, TGFβR1*, and *TGFβR2 *genes, although powerful, may miss mutations in the promoter region or in other noncoding sequences. Another consideration is that genes, such as *TGF*β*R1*and *TGF*β*R2 *may not harbour large deletions related to aortopathy with most of the reported *TGFβR1 *and *TGFβR2 *pathogenic mutations have been missense or splice site mutations [11], with all but one lying in the kinase domain of those genes [28]. Recently, a *de novo *14.6 Mb duplication on chromosome 9q22.32q31.2, comprising *TGF*β*R1 *was found in a 17-year-old male with dysmorphic features, suggestive of LDS [29]. In contrast, no large deletions of *TGF*β*R1*and *TGF*β*R2 *have ever been reported in patients with aortopathy, which concurs with the findings from our dataset. Finally, the lack of genotype-phenotype correlation in some patients could be due to uncharacterized genetic elements in other loci, and as such, better evaluated by technologies targeting the whole genome or selected high-yield genes involved on aortopathies and Marfan-like phenotypes, such as *MYH11, ACTA2, SLC2A10, NOTCH1 *and *FBN2 *genes [9].

## Conclusions

Our data expand the number of large *FBN1 *deletions, and emphasize the importance of screening for large genomic deletions in comprehensive genetic testing for connective tissue disorders featuring aortopathies, especially for those with classic phenotype of Marfan syndrome.

## Competing interests

The authors declare that they have no competing interests.

## Authors' contributions

LVF participated in the design of the study, carried out part of the MLPA experiments, and wrote the manuscript; WWD carried out part of the MLPA experiments and performed the sequencing experiments; TL performed the array-CGH experiments in two patients; PP assisted WWD with sequencing experiments; AFR and AY were responsible for diagnosis and management of patients, participated in the design of the study and revision of the final manuscript; PBT conceived the study, and participated in its design and coordination. All authors read and approved the final manuscript.

## Pre-publication history

The pre-publication history for this paper can be accessed here:

http://www.biomedcentral.com/1471-2350/12/119/prepub
